# Prognostic relevance of platelet lymphocyte ratio (PLR) in gastric cancer patients receiving immune checkpoint inhibitors: a systematic review and meta-analysis

**DOI:** 10.3389/fonc.2024.1367990

**Published:** 2024-06-07

**Authors:** Shufu Hou, Dandan Song, Yelei Zang, Ruiqi Hao, Linchuan Li, Jiankang Zhu

**Affiliations:** ^1^ Department of General Surgery, The First Affiliated Hospital of Shandong First Medical University, Jinan, China; ^2^ Key Laboratory of Metabolism and Gastrointestinal Tumor, The First Affiliated Hospital of Shandong First Medical University, Jinan, China; ^3^ Department of Neurology, Shandong Province Third Hospital, Jinan, China

**Keywords:** platelet-to-lymphocyte ratio, overall survival, progression-free survival, gastric cancer, immune checkpoint inhibitors

## Abstract

**Objectives:**

The prognostic relevance of the platelet-to-lymphocyte ratio (PLR) in gastric cancer (GC) patients undergoing immune checkpoint inhibitor (ICI) treatment remains unclear. This meta-analysis aimed to determine the prognostic impact of PLR in this specific patient cohort.

**Methods:**

We searched the PubMed, Cochrane Library, CNKI, and EMBASE databases, including literature published up to September 2023, to investigate the prognostic implications of PLR in patients with gastric cancer undergoing immune checkpoint inhibitor therapy. Outcome measures encompassed overall survival (OS), progression-free survival (PFS), objective response rate (ORR), and disease control rates (DCR).

**Results:**

Nine studies from seven articles comprising 948 eligible patients were selected. The results revealed a significant correlation between elevated PLR and poorer OS and progression-free survival (PFS) (OS: HR 1.67, 95% CI 1.39–2.00, p < 0.001; PFS: HR 1.51, 95% CI 1.29–1.76, p < 0.001). Subgroup analyses were performed to validate the robustness of the results. Moreover, a meta-analysis of four studies investigating the correlation between the PLR in gastric cancer (GC) patients and the objective response rate/disease control rate (ORR/DCR), showed no significant association between the PLR and ORR/DCR (ORR: RR = 1.01, p = 0.960; DCR: RR = 0.96, p = 0.319).

**Conclusions:**

This meta-analysis indicates that elevated PLR in GC patients undergoing ICI treatment is significantly linked to worse OS and PFS. Therefore, PLR can serve as a prognostic indicator of post-treatment outcomes in patients with GC receiving ICIs. Further prospective studies are required to assess the reliability of these findings.

**Systematic review registration:**

https://inplasy.com/, identifier INPLASY2023120103.

## 1 Introduction

Gastric cancer (GC) is a malignancy that accounts for 7.7% of cancer-related fatalities. Globally, it is the fifth most frequently diagnosed cancer and ranks third in cancer-related mortality (1, 2). Although the incidence rate has declined in recent years, there has been an increasing trend in younger populations. GC is characterized by insidious onset, rapid progression, high malignancy, and a poor prognosis (3). Most patients with gastric cancer are already in the advanced stages of the disease at the time of their initial consultation and have lost the opportunity for curative surgery. This implies that more intricate and comprehensive treatment approaches aimed at disease control, symptom alleviation, and improvement in survival quality may need to be developed. Although traditional treatments, such as chemotherapy, can improve overall survival and quality of life, their overall clinical efficacy is limited. The RAINBOW-Asia trial (4) reported a median overall survival of only 7.92 months in patients undergoing chemotherapy alone. Since the latter part of the 20th century, significant progress has been made in leveraging immunotherapy for the management of gastric cancer, particularly through the use of immune checkpoint inhibitors (ICIs). ICIs have become the mainstream treatment for many types of malignant tumors, revolutionizing cancer therapy (5–11). Blocking the PD-1/PD-L1 signaling pathway with immune checkpoint inhibitors plays a crucial role in identifying and preventing the escape of tumor cells. This mechanism improves the tumor microenvironment, stimulates the body’s immune system, enhances immune responses, and effectively eliminates tumor cells. Numerous studies have suggested ICI therapy as a promising therapeutic strategy. Whether used alone or in combination with other treatments such as chemotherapy, ICIs show significant potential in improving the survival of patients with advanced gastric cancer patients (12–15). Tumor mutational burden (TMB) (16), tumor-infiltrating lymphocytes (17), microsatellite instability (MSI) (18), and other tumor biomarkers have been widely studied as predictive biomarkers for PD-1/L1 inhibitor therapy. However, their application in clinical settings is limited because of the relatively complex detection process and the lack of consensus on numerical thresholds. Since Virchow first proposed a potential link between inflammation and tumors, abundant experimental and clinical data have unequivocally established a clear correlation between inflammatory responses and the initiation and progression of tumors (19–21). During chronic inflammation, inflammatory cells release numerous cytokines, chemotactic proteins, and other inflammatory mediators. By activating endogenous or exogenous signals, they alter the cellular microenvironment and promote tumor growth, proliferation, and metastasis. Systemic inflammation is a prominent manifestation of host-tumor interactions in cancer (22, 23) and has been acknowledged as the 7th hallmark of malignant tumors. Building upon this, more scholars have investigated the prognostic value of systemic inflammatory biomarkers, such as the neutrophil-to-lymphocyte ratio (NLR) and monocyte-to-lymphocyte ratio (MLR), in various types of cancer (24–26). The platelet-to-lymphocyte ratio (PLR) is associated with prognosis in many malignant tumors, including non-small cell lung cancer (NSCLC) and breast cancer. The platelet-to-lymphocyte ratio (PLR) has been proven to be associated with prognosis (27, 28). As a straightforward and easily accessible biomarker, PLR can be used to gauge the inflammatory status of the immune system. However, there is currently a lack of meta-analyses on the predictive significance of the PLR and its variations in gastric cancer (GC) patients undergoing immune checkpoint inhibitor (ICI) therapy. Therefore, we included relevant cohort studies to compare the prognosis and treatment response differences in patients with GC with different PLR values after ICI treatment. This study aimed to investigate the prognostic value of the PLR in this patient cohort.

## 2 Materials and methods

### 2.1 Search strategy

This systematic review and meta-analysis was carried out according to the guidelines outlined in the Preferred Reporting Items for Systematic Reviews and Meta-Analyses (PRISMA) (29). Two independent researchers systematically searched PubMed, Embase, CNKI and the Cochrane Library to identify relevant studies concerning the prognostic significance of the Platelet-to-Lymphocyte Ratio (PLR) in patients (GC) patients undergoing treatment with immune checkpoint inhibitors (ICIs). The search encompassed the period from the inception of these databases until September 30, 2023. It utilizes the following terms to investigate the predictive significance of PLR and ICIs in patients with gastric cancer: “Platelet-Lymphocyte ratio” or “Platelet-to-Lymphocyte ratio” or PLR, “gastric cancer” or “gastric adenocarcinoma”, and “PD-L1 inhibitors” or “immune checkpoint inhibitors” or “programmed cell death ligand-1 inhibitors” or “immunotherapy”. In addition to utilizing free search terms and Medical Subject Headings (MeSH) for searching within titles or abstracts, we screened the references of selected articles to ensure comprehensive retrieval.

### 2.2 Inclusion and exclusion criteria

Inclusion criteria: (1) Patients with gastric cancer confirmed through pathological examination, in advanced or locally advanced stages, and receiving immune checkpoint inhibitor (ICI) treatment; (2) Studies providing long-term survival data, including overall survival (OS) or progression-free survival (PFS); (3) Studies providing hazard ratio (HR) or relative risk (RR) with a 95% confidence interval (CI).

The exclusion criteria were as follows: (1) reviews, case reports, case series, conference abstracts, or commentaries; (2) studies lacking sufficient data; (3) nonclinical or nonhuman studies; and (4) data overlap or duplication.

### 2.3 Data extraction and quality assessment

Two researchers independently extracted data from the eligible studies, and discrepancies were resolved through discussions or consultations with a third researcher. The extracted data included the first author’s name, publication year, study country, study design, sample size, average or median age of the included patients, sex distribution, treatment modalities, survival analysis (including hazard ratios with corresponding 95% confidence intervals for overall survival and progression-free survival), and objective response rate (ORR) or disease control rate (DCR). The Newcastle-Ottawa Scale (NOS) was used to assess the quality of the studies, covering three aspects: selection (0–4 points), comparability (0–2 points), and outcome assessment (0–3 points). Two researchers individually scored each of the eight questions on these three aspects, with a total score range of 0–9 points. Studies with scores exceeding 6 points were considered high quality (30). The results of another bias risk assessment tool, ROBINS-I (31), can be found in the **Supplementary Materials**.

### 2.4 Statistical methods

This study used Stata SE (version 12.0; StataCorp, College Station, Texas, USA) for statistical analysis. The RR was used to assess the relationship between PLR, ORR, and DCR in patients with gastric cancer undergoing ICI treatment. HR and its associated 95% confidence interval (CI) were used to evaluate the potential association of PLR with OS and PFS. Cochran’s Q-test and I2 statistics were used to assess heterogeneity among the studies; based on this, an appropriate effect model was selected. A random-effects model was selected if I2 was > 50% or the p-value was < 0.10 (Q-test), indicating significant heterogeneity. Otherwise, a fixed effects model was used. Publication bias was evaluated by observing the symmetry of the funnel plot and utilizing methods such as Egger’s linear regression, Begg regression, where a P-value < 0.05 was considered indicative of publication bias. A sensitivity analysis was performed to explore the impact of different studies on OS and PFS. Subgroup analyses were conducted based on treatment methods, sample size, cutoff values, and analysis models to further investigate the sources of heterogeneity.

## 3 Results

### 3.1 Study selection and characteristics

**Figure 1:**
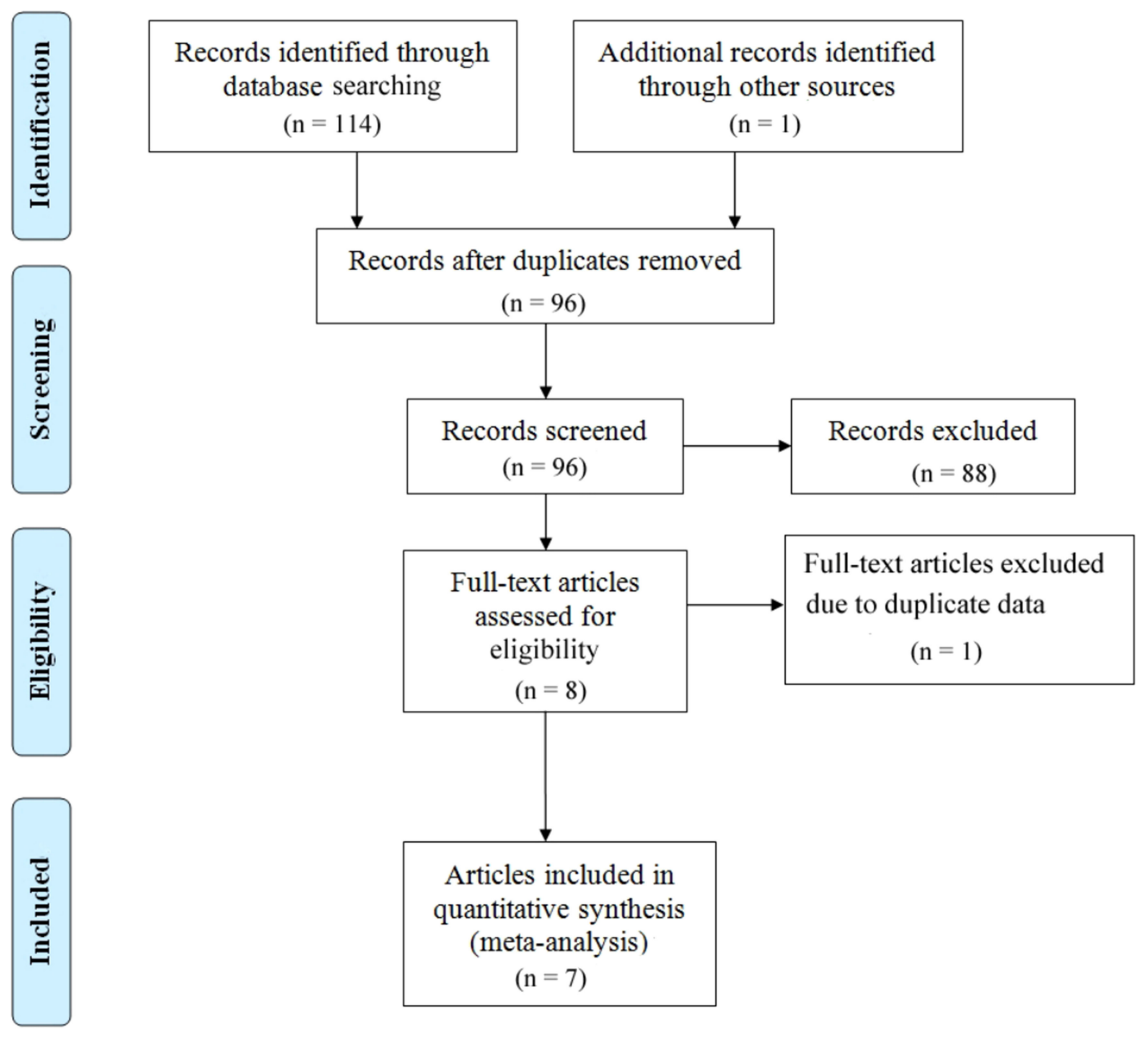
Prisma flowchart illustrating the literature selection process.

A comprehensive depiction of the literature selection process is shown in **Figure 1**. Following the previously outlines the search strategy and 114 articles were initially retrieved. After excluding duplicate studies, 96 studies were retained. After meticulous screening of titles and abstracts per the predefined inclusion and exclusion criteria, 88 studies were excluded. One study was excluded due to the unavailability of its full text. Ultimately, we identified seven articles covering nine observational cohort studies (32–38). **Table 1** summarizes the characteristics of the included studies. All these studies were published between 2021 and 2022, with eight studies conducted in China from six articles and one study from Japan. All seven articles were retrospective studies. The sample sizes ranged from 45 to 238, with a total of 948 patients. Among them, six studies employed ICIs alone, whereas the remaining two studies utilized combination chemotherapy, including ICIs. One study focused solely on Progression-Free Survival (PFS), whereas seven studies concurrently documented Overall Survival (OS) and PFS. Based on the Newcastle-Ottawa Quality Assessment Scale (NOS), the scores for all included studies ranged from 7 to 8, indicating relatively high data quality. The Newcastle-Ottawa Quality Assessment Scale (NOS) scores of all included articles are shown in **Table 2**. In addition, following the detailed guidelines of ROBINS-I (30), we employed the ROBINS tool to assess each study as if it were a hypothetical target randomized controlled trial, with deviations from this target trial considered biases. We evaluated seven bias domains, each categorized as “low,” “moderate,” “serious,” or “critical,” or marked as “no information” for judgment (**Supplementary Table 1**).

**Table 1:** Baseline characteristics of included studies.

Study, year	Country	Duration	Study design	Sample size	Age	Gender (M/F)	Follow-up (months)	Treatment	Cut-off	Survival outcome	Analysis	NOS
Chen 2021 (34)	China	2015–2019	Retrospective	139	median: 60 (51–67)	103/36	median: 23.8	ICIs	173.7	OS, PFS	M/M	8
Ruan 2021 (38)	China	NR	Retrospective	58	median: 60 (52–66)	41/17	median: 4.5	ICIs	267.21	OS, PFS	M/U	7
Gou 2022 (35)	China	2016–2021	Retrospective	237	median: 59 (34–86)	109/128	NR	ICIs	139.41	OS, PFS	M/M	7
Hayano 2022 (36)	Japan	2018–2021	Retrospective	70	median: 71 (23–87)	48/22	NR	ICIs	152.5	PFS	U	7
Pan 2022 (32)	China	2014–2021	Retrospective	238	median: 58 (18–86)	176/62	NR	ICIs	163.63	OS, PFS	U/U	7
Qu 2022 1 (37)	China	2019–2021	Retrospective	53	NR	NR	median: 17.5	ICIs	243.33	OS, PFS	M/U	8
Qu 2022 2 (37)	China	2019–2021	Retrospective	53	NR	NR	median: 15.9	ICIs	243.33	OS, PFS	M/M	8
Wan 2022 1 (33)	China	2017–2020	Retrospective	45	median: 64 (37–74)	35/10	median: 27.3	ICIs+chemotherapy	214.08	OS, PFS	U/U	8
Wan 2022 2 (33)	China	2017–2020	Retrospective	55	median: 65 (32–81)	42/13	median: 15.3	ICIs+chemotherapy	214.08	OS, PFS	U/U	8

M, male; F, female; NR, not report; ICIs, immune checkpoint inhibitors; OS, overall survival; PFS, progression-free survival; U, univariate; M, multivariate; NOS, Newcastle-Ottawa Scale.

**Table 2:** Newcastle-Ottawa Scale (NOS) for quality assessment.

Studies	Selection				Comparability	Outcome			Scores
	A	B	C	D	E	F	G	H	
Chen 2021 (34)	★	★	★	★	★★	★	★	–	8
Ruan 2021 (38)	★	★	★	★	★★	★	–	–	7
Gou 2022 (35)	★	★	★	★	★★	★	–	–	7
Hayano 2022 (36)	★	★	★	★	★★	★	–	–	7
Pan 2022 (32)	★	★	★	★	★★	★	–	–	7
Qu 2022 1 (37)	★	★	★	★	★★	★	★	–	8
Qu 2022 2 (37)	★	★	★	★	★★	★	★	–	8
Wan 2022 1 (33)	★	★	★	★	★★	★	★	–	8
Wan 2022 2 (33)	★	★	★	★	★★	★	★	–	8

A study may receive a maximum of one star for each numbered item in the Selection and Outcome categories. A maximum of two stars may be given for Comparability, as directed by the NOS.

★: It stands for one point; ★★: It stands for two points.

### 3.2 Association of PLR with OS and PFS

**Figure 2:**
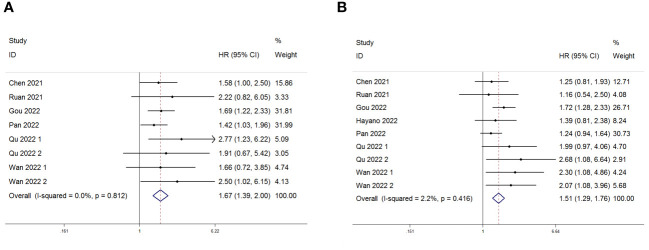
Forest plot for the association between Platelet-to-Lymphocyte Ratio (PLR) expression and **(A)** overall survival (OS) and **(B)** progression-free survival (PFS) in gastric cancer patients receiving Immune Checkpoint Inhibitors (ICIs).

Eight studies, including 878 patients, investigated the relationship between PLR and OS in patients with GC receiving Immune Checkpoint Inhibitors (ICIs). Heterogeneity testing indicated non-significant heterogeneity (P=0.812 > 0.1, I^2^ = 0.0% < 50%), suggesting that the fixed-effects model was suitable for the meta-analysis. The combined Hazard Ratio (HR) and its 95% Confidence Interval (CI) were as follows: HR=1.67, 95% CI=1.39–2.00, P<0.001, indicating that a higher PLR predicts poorer OS in GC patients undergoing ICIs (**Figure 2A**). In addition, nine studies involving 948 patients explored the relationship between the PLR and Progression-Free Survival (PFS) in patients with GC receiving ICIs. Heterogeneity testing revealed no significant heterogeneity (P=0.416 > 0.1, I2 = 2.2%, < 50%). The combined results showed that HR=1.51, 95% CI= 1.29–1.76, and P<0.001, indicating that higher PLR values are associated with worse PFS in GC patients (**Figure 2B**). Subgroup analyses, considering treatment (ICIs or ICIs + chemotherapy), sample size (≥100 or <100), cutoff (>214.08 or ≤214.08), and analysis (multivariate or univariate), consistently revealed that a higher PLR predicts poorer OS and PFS in patients (**Tables 3**, **4**).

**Table 3:** Subgroup analysis evaluating the prognostic significance of PLR for OS in gastric cancer patients treated with ICIs.

Subgroup	NO. of studies	HR (95% CI)	P	Heterogeneity		Model
				I2 (%)	Ph	
Treatment						
ICIs	6	1.64 (1.35–1.98)	<0.001	0	0.715	Fixed
ICIs+chemotherapy	2	2.01 (1.09–3.71)	0.026	0	0.514	Fixed
Sample size						
>100	3	1.55 (1.27–1.91)	<0.001	0	0.747	Fixed
<100	5	2.20 (1.47–3.29)	<0.001	0	0.925	Fixed
Cuf-off						
≥214.08	5	2.20 (1.47–3.29)	<0.001	0	0.925	Fixed
<214.08	3	1.55 (1.27–1.91)	<0.001	0	0.747	Fixed
Analysis						
Univariate	3	1.53 (1.15–2.03)	0.004	0	0.495	Fixed
Multivariate	5	1.77 (1.40–2.24)	<0.001	0	0.789	Fixed

**Table 4:** Subgroup analysis evaluating the prognostic significance of PLR for PFS in gastric cancer patients treated with ICIs.

Subgroup	NO. of studies	HR (95% CI)	P	Heterogeneity		Model
				I2 (%)	Ph	
Treatment						
ICIs	7	1.45 (1.23–1.7)	<0.001	0	0.445	Fixed
ICIs+chemotherapy	2	2.17 (1.32–3.54)	0.002	0	0.835	Fixed
Sample size						
>100	3	1.41 (1.17–1.69)	<0.001	29.3	0.243	Fixed
<100	6	1.77 (1.33–2.35)	<0.001	0	0.617	Fixed
Cuf-off						
≥214.08	5	1.94 (1.39–2.71)	<0.001	0	0.652	Fixed
<214.08	4	1.40 (1.18–1.67)	<0.001	0	0.418	Fixed
Analysis						
Univariate	6	1.43 (1.17–1.76)	<0.001	0	0.425	Fixed
Multivariate	3	1.61 (1.27–2.04)	<0.001	26.2	0.258	Fixed

### 3.3 Publication bias

**Figure 3:**
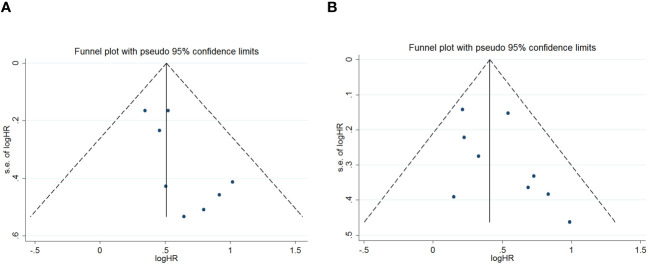
Funnel plots are utilized to assess the presence of publication bias in **(A)** overall survival (OS) and **(B)** progression-free survival (PFS).

**Figure 4:**
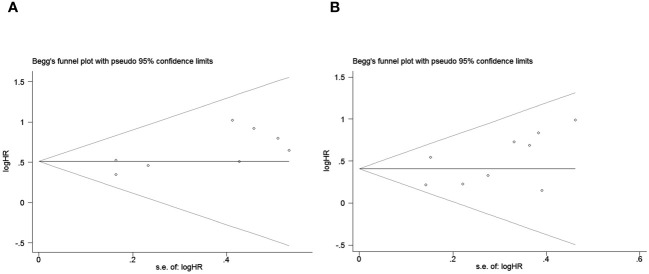
Publication bias test. **(A)** Begg tests for OS, p = 0.266; **(B)** Begg tests for PFS, p = 0.118.

**Figure 5:**
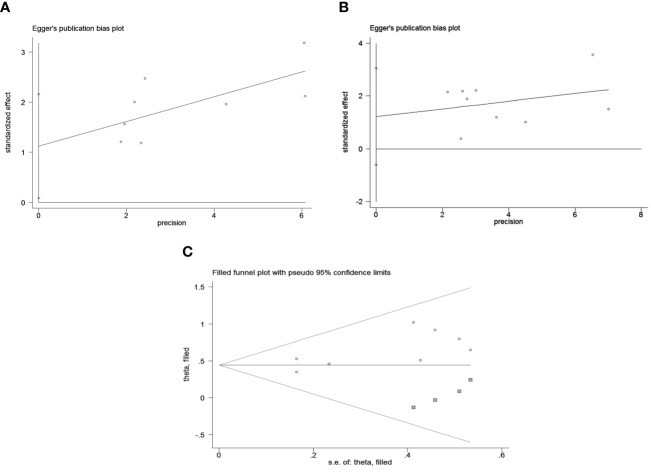
Publication bias test. **(A)** Egger’s test for OS, p = 0.037; **(B)** Egger’s test for PFS, p = 0.158; **(C)** The funnel diagram corrected by the cut-and-patch method in OS.

Publication bias was assessed using funnel plots, Egger’s linear regression and Begg regression. Funnel plots for Progression-Free Survival (PFS) exhibited favorable symmetry (**Figure 3A**). However, the symmetry of the funnel plot for overall survival (OS) is not as effective as that for progression-free survival (PFS) (**Figure 3B**).The Begg tests indicated no significant publication bias for OS or PFS (OS, p = 0.266; PFS, p = 0.118; **Figure 4**). The outcomes from the Egger (OS, p = 0.037; **Figure 5A**; PFS, p = 0.158; **Figure 5B**) tests revealed a probable publication bias inside the relevant OS investigations. Nevertheless, we obtained symmetrical funnel plots applying the cut-and-patch method (**Figure 5C**), showing that the results were still statistically significant and robust, without substantial interferences from publication bias, with an HR of 1.559 (95% CI: 1.316–1.847).

### 3.4 Sensitivity analysis

**Figure 6:**
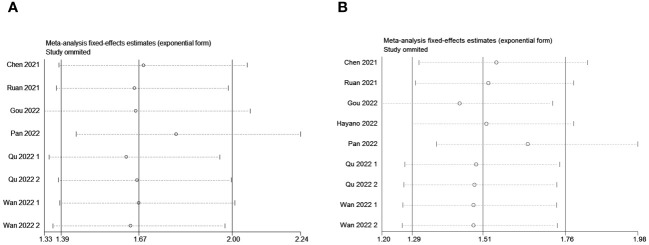
Sensitivity analysis for the pooled results between PLR and **(A)** OS and **(B)** PFS.

Sensitivity analysis revealed that no individual study significantly influenced the observed effect size of the association between the PLR and OS or PFS. In this study, removing a single article did not result in significant changes, indicating the reliability of the results (**Figure 6**).

### 3.5 PLR and ORR/DCR association

**Figure 7:**
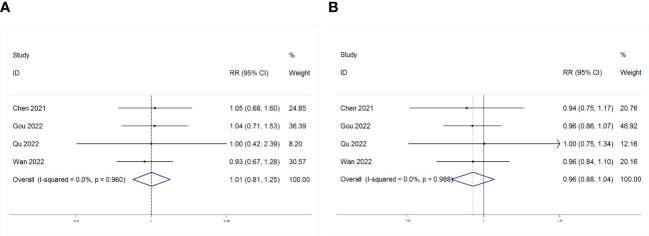
Forest plot for the association between PLR and **(A)** objective response rate (ORR) and **(B)** disease control rate (DCR).

As depicted in **Figure 7**, four studies examined the association between PLR and treatment outcomes (ORR or DCR) in patients (GC) undergoing Immune Checkpoint Inhibitors (ICIs). PLR showed no significant correlation with ORR or DCR (ORR: RR = 1.01, p = 0.960; DCR: RR = 0.96, p = 0.319).

## 4 Discussion

Gastric cancer (GC) is the leading cause of cancer-related fatalities worldwide. Despite notable progress in GC treatment in recent years, persistently high rates of recurrence and mortality prevail (39, 40). The primary challenge stems from the nonspecific nature of early-stage GC detection, with most patients being diagnosed during the advanced stages of cancer. Thus, the identification of biomarkers capable of predicting post-treatment prognosis is of paramount significance for GC treatment. Lymphocytes and platelets play pivotal roles in the systemic inflammatory response, demonstrating crucial functions in tumor development, infiltration, and metastasis (19, 41, 42). Tumor cells employ various mechanisms to activate platelets, leading to the direct release of factors such as IL-1, thrombin, and endothelin, thereby promoting tumor angiogenesis and enhancing tumor migration and dissemination (43). Platelet aggregates encapsulate circulating tumor cells, bolstering their ability to evade host immune attacks (44). Lymphocytes show robust antitumor activity and effectively hinder tumor cell proliferation and metastasis throughout cancer progression (45). A reduced lymphocyte count could result in an inadequate immune response, consequently exerting an adverse impact on the prognosis of patients with solid tumors (46). Platelet-to-lymphocyte ratio (PLR), characterized by its cost-effectiveness and ready accessibility, has garnered widespread attention among scholars. Numerous studies have demonstrated an association between PLR and the prognosis of patients with gastric cancer, including overall survival and progression-free survival (47–50). Tomás and Tiago Cruz (51) affirmed that patients with an elevated PLR have a heightened risk of progression and mortality. Hirahara (52) identified the predictive potential of the combined NLR and platelet-to-lymphocyte ratio (PLR) for treatment outcomes and prognosis in patients with advanced gastric cancer. Tang, Cheng (53) corroborated a substantial association between high PLR and a diminished objective response rate (ORR) in advanced gastric cancer patients following neoadjuvant chemotherapy. Although the Platelet-to-Lymphocyte Ratio has shown prognostic value in diverse cancers, research specifically investigating its prognostic significance in patients undergoing immune checkpoint inhibitor (ICI) therapy is still somewhat limited. A recent retrospective study by Wan M (33), et al. suggested that the PLR and systemic inflammation markers are associated with PFS in patients with gastric cancer receiving immune checkpoint inhibitors (ICIs), while showing no correlation with overall survival (OS). Through univariate and multivariate analyses, Gou (35) concluded that PLR was an independent prognostic factor for both OS and PFS. Additionally, findings from Ziting Qu’s study (37) indicated that PLR acts as an independent prognostic factor for overall survival (OS) in patients with advanced gastric cancer (AGC) undergoing first-line immunotherapy and for PFS in patients undergoing second-line or subsequent immunotherapy. Chen Y (34) suggested a correlation between high PLR, low DCR and ORR. To address this confusion and fill the knowledge gap, we conducted a meticulous meta-analysis of data from nine relevant trials involving 948 patients across two nations. Our study aimed to elucidate whether PLR values could predict the survival outcomes of patients with gastric cancer undergoing immune checkpoint inhibitor (ICI) treatment. In comparison with these studies, our research is the first meta-analysis to incorporate PLR into the analysis of patients with gastric cancer undergoing ICI treatment. Our analysis revealed a discernible trend; elevated PLR values were significantly correlated with reduced survival rates, indicating a significant association between increased PLR and diminished OS and PFS. The combined hazard ratio (HR) for PLR and OS was 1.67, and for PLR and PFS, it stood at 1.51. Furthermore, our subgroup analyses indicated that, irrespective of treatment type (ICIs or ICIs + chemotherapy), sample size (≥100 or <100), cutoff value (>214.08 or ≤214.08), and analysis type (multivariate or univariate), higher PLR values were consistently associated with poorer OS and PFS. The sensitivity analysis and assessment of publication bias also demonstrated the robustness of the results of this meta-analysis. However, there was no statistically significant correlation between PLR and treatment response (ORR/DCR) (ORR: RR = 1.01, p = 0.960; DCR: RR = 0.96, p = 0.319). It is crucial to note that certain limitations should not be overlooked when interpreting our findings. Firstly, the reduction in evidence quality is attributed to the observational and retrospective nature of all included studies, as well as the small sample sizes. Secondly, immune-related adverse events (irAEs) are an inevitable issue that needs to be discussed in the context of immunotherapy. The use of immune checkpoint inhibitors (ICIs) increases the occurrence of irAEs, leading to off-target immune activation and inflammatory reactions. Severe irAEs may necessitate the temporary suspension or dose adjustment of immunotherapy agents, and severe or fatal toxicities pose significant challenges to immunotherapeutic approaches. Due to limitations in the original literature, this study only elucidates the relationship between PLR and overall survival (OS) and progression-free survival (PFS), without revealing the correlation between PLR and irAEs (54, 55). Lastly, since the synthesis of samples is based on studies meeting search criteria, selection bias may be present.

## 5 Conclusions

For gastric cancer patients receiving treatment with immune checkpoint inhibitors (ICI), our meta-analysis showed a significant correlation between a higher Platelet-to-Lymphocyte Ratio (PLR) and worse Overall Survival (OS) and Progression-Free Survival (PFS). PLR is a promising prospective biomarker for the prognostication of patients with gastric cancer receiving ICIs. However, considering the limitations of our meta-analysis, larger-scale, multicenter, high-quality prospective trials are needed to validate our results.

## Data availability statement

The original contributions presented in the study are included in the article/**Supplementary Material**. Further inquiries can be directed to the corresponding author/s.

## Author contributions

SH: Writing – review & editing, Writing – original draft, Methodology, Formal analysis, Conceptualization. DS: Writing – review & editing, Writing – original draft, Methodology, Conceptualization. YZ: Writing – original draft, Project administration, Data curation. RH: Writing – original draft, Project administration, Data curation. LL: Writing – review & editing, Formal analysis. JZ: Writing – review & editing, Supervision.
