# Supplementary material for: Prognostic relevance of platelet lymphocyte ratio (PLR) in gastric cancer patients receiving immune checkpoint inhibitors: a systematic review and meta-analysis
Source: Front Oncol. 2024 Jun 7;14:1367990. doi: 10.3389/fonc.2024.1367990 (PMC11190700; doi:10.3389/fonc.2024.1367990)
Supplement: Supplementary file 1 [file Table_1.pdf]

Supplementary Table 1. Risk Of Bias In Non-randomized Studies - of Interventions (ROBINS-I) for quality assessment

Studies	Bias due to confounding	Bias in selection of participants	Bias in classification of interventions	Bias due to deviations from intended interventions	Bias due to missing data	Bias in measurement of outcomes	Bias in selection of the reported result	Overall ROB judgment
Chen 2021	Low	Low	Low	Low	Low	Low	Low	Low
Ruan 2021	Low	Low	Low	Low	Low	Low	Low	Low
Gou 2022	Low	Low	Low	Low	Low	Low	Low	Low
Hayano 2022	Critical	Low	Low	Low	Moderate	Low	Low	Critical
Pan 2022	Critical	Low	Low	Low	Low	Low	Low	Critical
Qu 2022 1	Low	Low	Low	Low	Low	Low	Low	Low
Qu 2022 2	Low	Low	Low	Low	Low	Low	Low	Low
Wan 2022 1	Critical	Low	Low	Low	Low	Low	Low	Critical
Wan 2022 2	Critical	Low	Low	Low	Low	Low	Low	Critical
